# Draft genome sequence of *Lacticaseibacillus paracasei* strain LPML01 isolated from the human gut

**DOI:** 10.1128/mra.00806-25

**Published:** 2025-12-05

**Authors:** Hasan Mahfuz Reza, Abu Reza, Md. Ashikuzzaman Antor, Zinia Afrin, Tonima Enam, Mahadi Hasan, Afsana Binte Yunus, Md. Shahedur Rahman, Mohammad Abu Hena Mostofa Jamal

**Affiliations:** 1 Department of Biotechnology and Genetic Engineering, Faculty of Biological Sciences, Islamic University 247297, Kushtia, Bangladesh; 2 Laboratory of Medical and Environmental Biotechnology, Islamic University 247297, Kushtia, Bangladesh; 3 Department of Pharmacy, Faculty of Biological Sciences, Islamic University 247297, Kushtia, Bangladesh; 4 Department of Genetic Engineering and Biotechnology, Faculty of Biological Sciences and Technology, Jashore University of Science and Technology 421984, Jashore, Bangladesh; 5 Bioinformatics and Microbial Biotechnology Laboratory, Jashore University of Science and Technology 421984, Jashore, Bangladesh; University of Maryland School of Medicine, Baltimore, Maryland, USA

**Keywords:** Whole genome sequence, *Lacticaseibacillus paracasei*, human gut, probiotics

## Abstract

This study reports the draft genome sequence of *Lacticaseibacillus paracasei* LPML01, a strain of a well-known probiotic species isolated in Bangladesh in 2024. The genome length is 3,061,259 base pairs (bp) with a GC content of 46.13 %.

## ANNOUNCEMENT

*Lacticaseibacillus paracasei* is a well-known probiotic species with potential health-promoting properties (1). This study reports the draft genome sequence of *Lacticaseibacillus paracasei* LPML01, a strain isolated from a human gut sample in 2024, to investigate its probiotic characteristics and genetic features. Because it originates from the human intestinal environment, this strain may be a safe, naturally adapted candidate for probiotic applications.

The stool sample was collected in a sterile collection tube from the 250-bed general hospital, Kushtia. The sample was homogenized in sterile PBS, and serial 10-fold dilutions were plated on MRS agar to isolate lactic acid bacteria, which were incubated for 24 h at 37°C. Although MRS agar is not specifically selective for *L. paracasei*, it is commonly used for the enrichment and preliminary isolation of *Lactobacillus* species (2). A pure culture was prepared from a single colony on a spread culture plate, under the same culture conditions previously mentioned. A single colony from the pure culture was inoculated into MRS broth and incubated overnight at 37°C and 120 rpm. Genomic DNA was extracted from 1 mL broth culture using the CDC PulseNet Total DNA Extraction protocol (https://www.aphl.org/programs/global_health/Documents/PNL33_DNA_Extraction_and_Quality.pdf), which was performed with the Qiagen DNeasy kit to achieve optimal DNA purity. DNA quality was assessed using a Qubit 4.0 Fluorometer and a Nanodrop spectrophotometer, with the Qubit Buffer used at a 1:200 ratio.

Whole-genome sequencing was performed on the Illumina NextSeq 550 platform (paired-end 2 × 150 bp) following the manufacturer’s instructions. During library preparation, genomic DNA was enzymatically sheared via tagmentation using the Illumina DNA Prep Reagent Kit, and fragments (200–300 bp) were size-selected with AMPure XP beads. The sequencing library was prepared and adapter-tagged using an automated liquid handler (epMotion 5075). Raw reads were demultiplexed using bcl2fastq v2.20.0 (https://support.illumina.com/sequencing/sequencing_software/bcl2fastq-conversion-software.html).

Raw reads were trimmed with Sickle v1.3 using paired-end trimming set to a length of 20 (3), then assembled with SPAdes v4.0.0 (4). Pilon v1.24 polished the assembly (5). The assembled genome was submitted to PATRIC under BV-BRC v3.54.6 (https://www.bv-brc.org) for detailed genomic analysis (6). A whole-genome-based taxonomic analysis was performed on the Type (Strain) Genome Server (https://tygs.dsmz.de) (7), with 16S and whole-genome phylogenetic analyses confirming *L. paracasei* as the species of the isolated strain, designated as LPML01. Default parameters were used for all software unless otherwise specified.

The genome length of *L. paracasei* strain LPML01 is 3,061,259 base pairs, with a GC content of 46.3%. The assembly comprises 37 contigs. The assembled genome was annotated using PATRIC and PGAP v6.10 annotation pipelines (8, 9). Both annotation systems identified 4 rRNA genes; however, PGAP predicted 2,904 coding sequences (CDSs), whereas the PATRIC system identified 3,109 CDS regions. These minor differences likely result from variations in the algorithms, gene prediction criteria, and reference databases used by the two annotation platforms. For instance, PGAP relies on NCBI’s curated RefSeq database, whereas PATRIC integrates multiple reference sources (10). A summary of the annotation results is provided in Table 1.

**TABLE 1:** Genomic features of *L. paracasei* strain LPML01

Sequencing summary	
Coverage	56.86 ×
Raw reads	1,306,989
GC content	45.3%
Size	163.4 MB

Assembly report (PATRIC)	
Contigs	37
GC content	46.13
Contig L50	3
Genome length	3,061,259
Contig N50	339,034

Genome report (PATRIC)	
Coarse consistency (%)	99.9
Fine consistency (%)	98.4
Completeness (%)	100
Contamination (%)	0

Annotation report		
Tool used	PGAP	PATRIC
Contigs	37	37
Bases	3,061,259	3,061,259
CDS	2,904	3,109
ncRNA	3	0
rRNA	4	4
Repeat regions	Not detected	21
tRNA	48	48

## Data Availability

This Whole Genome Shotgun project has been deposited in GenBank under accession number JBNJSA000000000.1 (https://www.ncbi.nlm.nih.gov/nuccore/JBNJSA000000000.1). The raw sequence reads are available in the SRA database under accession number SRR31300363 (https://www.ncbi.nlm.nih.gov/sra/SRR31300363). Both data are under BioSample number SAMN48190442 (https://www.ncbi.nlm.nih.gov/biosample/SAMN48190442) and BioProject number PRJNA1256676 (https://www.ncbi.nlm.nih.gov/bioproject/PRJNA1256676). The genome assembly is available under the accession ASM5011393v1 (https://www.ncbi.nlm.nih.gov/datasets/genome/GCF_050113935.1/). The assembled genome has been submitted to PATRIC and is available under genome ID 1597.2345 (https://www.bv-brc.org/view/Genome/1597.2345).
